# Emergency TREPP for Strangulated Inguinal Hernia Repair: A Consecutive Case Series

**DOI:** 10.1055/s-0040-1705171

**Published:** 2020-03-31

**Authors:** T.L.R. Zwols, W.L. Akkersdijk, W.J.V. Bökkerink, C.S. Andeweg, J.P.E.N. Pierie, G.G. Koning

**Affiliations:** 1 Department of Surgery, Medical Centre Leeuwarden, Leeuwarden, The Netherlands; 2 Department of Surgery, St Jansdal Hospital, Harderwijk, The Netherlands; 3 Department of Surgery, Gelderse Vallei Hospital, The Netherlands; 4 Postgraduate School of Medicine, University Medical Centre Groningen, Groningen, The Netherlands; 5 Department of Surgery, Ikazia Hospital, Rotterdam, the Netherlands

**Keywords:** strangulated inguinal hernia, incarcerated, TREPP, open preperitoneal, extraperitoneal, emergency

## Abstract

**Background** Patients with strangulated inguinal hernia (SIH) require emergency surgical treatment. International guidelines do not specify the surgical technique of preference. Frequently, an open anterior approach such as the Lichtenstein technique is used.

The TransREctus sheath Pre-Peritoneal (TREPP) technique is an alternative, open posterior approach, which has shown promising results in the elective treatment of inguinal hernias. This study aims to evaluate the feasibility and safety of the TREPP technique in the emergency setting of SIHs.

**Materials and Methods** After medical ethical approval was warranted, all consecutive patients, who underwent emergency TREPP (e-TREPP) at a high-volume hernia institute, were retrospectively included from 2006 up to and including 2016. Data retrieved from the electronic patient files were combined with the findings during a long-term outcome physical investigation at an outpatient department visit. e-TREPP was, prior to the start of the study, defined as TREPP performed immediately at the operation room.

**Results** Thirty-three patients underwent e-TREPP for SIH. Ten patients were clinically evaluated, ten patients were deceased, nine patients could not be contacted, and four patients did not or could not consent. Of the ten deceased patients, one patient died perioperatively due to massive aspiration followed by cardiac arrest. Nine patients died due to other causes. Two patients developed a recurrence after (after 13 days and 16 months respectively). Two patients were surgically treated for a wound infection (mesh removal in one). No patient reported chronic postoperative inguinal pain.

**Conclusion** e-TREPP in experienced hands seems feasible and safe (Level of Evidence 4) for the treatment of patients with strangulated inguinal hernia, with percentages of postoperative complications comparable to other techniques.

Inguinal hernia repair is one of the most frequently performed surgical procedures in the Netherlands (∼30,000 per year). 1 Surgery is generally recommended in symptomatic inguinal hernia, but asymptomatic inguinal hernias can likewise lead to life-threatening complications. 2 3 Patients with an inguinal hernia risk developing a strangulated inguinal hernia (SIH). The rate of strangulation is estimated to be 0.3 to 3% of inguinal hernias per year. 4 Especially in femoral hernias, the risk of strangulation is increased, 5 Level of Evidence (LoE) 2B. 6 The definition of a SIH is an inguinal hernia that is nonreducible (incarcerated) and shows symptoms of strangulation (vascular occlusion [or nonperfusion] by impingement of the hernia content) and/or ileus. 3 If untreated, a SIH is life-threatening. Symptoms of a SIH include a sudden pain that quickly intensifies; nausea; vomiting (or both); fever; a groin bulge that turns red, purple, or dark; and clinical signs of bowel obstruction. 2

In the case of a (suspected) SIH, emergency surgery is needed to prevent bowel ischemia, necrosis, and perforation. The current guidelines recommend a tailored surgical approach for this situation, considering the fact that no randomized studies or systematic reviews exist in which a specific surgical technique is recommended. 3 The guideline recommends “timely” surgery, with literature suggesting that treatment after 12 hours after onset of symptoms significantly increases morbidity and mortality. 3

The TransREctus sheath Pre-Peritoneal (TREPP) technique was developed in 2006 as an open preperitoneal technique for inguinal hernia surgery reducing the rate of chronic postoperative inguinal pain (CPIP). 7 The TREPP has gained popularity in the Netherlands and seems to be a promising technique for both primary and recurrent inguinal hernias. 8 9 At the St Jansdal Hospital (Harderwijk, the Netherlands), the TREPP is the primary technique used for surgical repair of inguinal hernia, elective or acute. In the acute setting, the TREPP provides the possibility of opening the peritoneum, inspection of hernia sac contents, and potential resection of necrotic tissue, while still preserving the advantages of mesh location and complete overview as described for the elective setting. 7 To ensure the safety and efficacy of emergency TREPP (e-TREPP) critical evaluation is needed in this stage of development. 10 This study aims to evaluate the feasibility of the e-TREPP technique for patients with a SIH.

## Materials and Methods

### Patients

Prior to the start of the study, the protocol was registered (NCT03935490) 11 and approved by the official Medical Ethical Board (METC 2019/161). In line with literature, a SIH was defined as an inguinal hernia that is nonreducible (incarcerated) and shows symptoms of strangulation (vascular disorders of the hernia content) and/or ileus. 3 For this study, all consecutive patients who underwent e-TREPP for SIH were included. Screening for eligibility was done by reading the surgical reports of all patients who underwent a TREPP from January 2006 until December 2016. All operation records were evaluated to assess if an e-TREPP was performed indeed. Inclusion criteria were diagnosis with suspected SIH and treatment with e-TREPP. Exclusion criteria were other surgical techniques or postponed surgery (elective). The selected patients were contacted and informed about the aim of this study. After written informed consent, they were invited for a visit at the outpatient department (OPD) for physical examination. A home visit by the investigator was offered when an OPD visit was not possible. All data of patients who underwent e-TREPP were analyzed with longest follow-up that could be retrieved. If patients died during follow-up, all data were analyzed until moment of death.

### Surgical Technique

The e-TREPP operations were performed by three experienced hernia surgeons in line with the TREPP mesh repair that has been described previously for primary as well as recurrent inguinal hernia. 7 8 9 In summary, a 4 to 5 cm (cm) transverse incision is made about 1 cm above the localization of the deep inguinal ring. The anterior rectus sheath is opened. After retracting the rectus muscle medially, the preperitoneal space is bluntly dissected and a complete overview can be achieved. All possible (recurrent and/or persistent) hernia orifices can be visualized such as a direct, indirect, pantaloon, and/or femoral hernia. The strangulated hernia content was carefully observed to assess its recovery, both in bowel movement and in color. The surgeon decided if bowel segment resection was necessary and, if no perforation nor spill was present, a self-expandable mesh was positioned in the preperitoneal space (PolySoft hernia patch “Large,” Bard, currently part of BD Medical, the Netherlands). Due to the “upstream principle,” no mesh fixation is necessary. 7 12

### Data Collection

Outcomes regarding benefits and harm of e-TREPP for SIH were retrieved from the digital patient records and checked for accuracy during the patient interview at the OPD or during home visit. Demographic data regarding age at time of operation and gender were extracted from the electronic patient files. Further data collection involved perioperative outcomes of the e-TREPP for SIH (such as presence of irreversible bowel ischemia and/or need for bowel segment resection, duration of surgery, European Hernia Society classification, type of anesthesia, and any reported damage to vessels, nerves, or spermatic cord) and postoperative adverse events such as wound infection, hematoma, seroma, mesh infection, urinary retention, length of hospital stay, recurrence, CPIP, reoperation, persisting numbness, and procedure-related mortality. Patients were asked to categorize any complaints in either “discomfort” or “pain.” Discomfort was defined as any unpleasant but nonpainful feeling that “irritated” or “annoyed” the patient. CPIP was defined as any form of pain present after 3 months postoperatively, in compliance with international guidelines. 12 Patients were asked to fill in the visual analog scale (VAS) for pain at rest and during physical activity. The study design (consecutive case series, retrospective analysis, long-term clinical prospective follow-up) precluded a relevant measurement of health-related quality of life. Results of this study are reported in line with the Preferred Reporting of CasE Series in Surgery guideline. 13

## Results

After a search on urgent inguinal hernia repairs in the period 2006 up to and including 2016 in our center, a list of 221 operations was comprised, performed on 208 different patients. One-hundred seventy-five patients didn't fit the inclusion criteria (either elective [ *n* = 163] or semielective, i.e., next day [ *n* = 12], or different surgical technique [ *n* = 3]).

Thirty-three patients were diagnosed with a SIH for which an e-TREPP was performed immediately (within 12 hours after onset of symptoms). The operation reports and patient files of all 33 patients were evaluated and short-term complications were noted. Out of the total 33 operation reports, 26 report incarcerated or strangulated tissue in the hernia sack; in the other seven operation reports, there was either no sign of strangulation visible during surgery or the inguinal hernia repositioned during start of anesthesia. Of the 26 reports of visible incarcerated or strangulated tissue, bowel resection took place in seven patients. In the other 19 cases, either only nonbowel tissue was strangulated (e.g., appendix epiploica, lipoma, mesenterium) or the tissue appeared vital after release, for which no resection was required. Baseline characteristics and both short-term and long-term results are shown in Tables 1 and 2 .

**Table 1:** Baseline characteristics of included e-TREPP patients

	*n* = 33
Age a (y)	67 (range: 23–97)
Gender	
Male	23 (69.7%)
Female	10 (30.3%)
Side of SIH	
Left	10 (30.3%)
Right	23 (69.7%)
Duration of surgery (minutes)	32 (range: 12–58)
Anesthesia type	
Spinal	25 (75.8%)
General	8 (24.2%)
Type of hernia, when noted b	
Medial	5 (26.3%)
Lateral	9 (47.4%)
Femoral	5 (26.3%)

Abbreviations: e-TREPP, TransREctus sheath Pre-Peritoneal; SIH, strangulated inguinal hernia.

a At time of operation.

b Based on European Hernia Society (EHS) Classification. 14 Noted in 19/33 operation records due to introduction in 2007.

**Table 2:** Short-term adverse events of e-TREPP for strangulated inguinal hernia repair

	*n* = 33
Short-term (<30 d):	
Mortality a	1
Wound infection	1
Abscess	1
Mesh removal	1
Bleeding	0
Hematoma	2
Early recurrence	1
Ileus (reoperation needed)	1

Abbreviation: e-TREPP, emergency TransREctus sheath Pre-Peritoneal.

a Caused by massive aspiration followed by absence of cardiac activity.

**Table 3:** Long-term adverse events of e-TREPP for strangulated inguinal hernia repair

	*n* = 10 a
Long-term (>30 d):	
Recurrence b	1
Discomfort in groin area (no pain)	1
Hypersensitivity in groin area c	1

Abbreviation: e-TREPP, emergency TransREctus sheath Pre-Peritoneal.

a Long-term follow-up was performed in 10/33 patients.

b Patient developed recurrence after 16 months, treated with re-TREPP. 7

c During moments of increased abdominal pressure.

### (Serious) Adverse Events

Because of the strangulation, no patient was treated in day care. During short-term follow-up (<30 days), one patient developed a recurrence after 13 days, which was repaired by means of the TREPP technique (re-TREPP 9 ) ( Table 3 ). Other interventions were necessary in three patients: one patient developed an ileus for which reoperation was needed and two patients were surgically treated for an infection. One of the two patients developed an abscess, for which the mesh was removed. The other patient only had a superficial wound infection and the mesh was not involved.

An attempt was made to contact all 33 patients for long-term follow-up. Nineteen patients were lost in follow-up due to various reasons, such as death ( *n* = 10, not e-TREPP related), living abroad ( *n* = 1), moved away ( *n* = 2), didn't respond ( *n* = 6). The remaining 14 patients responded, of which 10 patients agreed to a visit to the OPD. The other four patients declined to cooperate ( *n* = 3) or were incapable ( *n* = 1, due to a mental handicap). All data taken from the patients' files were verified when the patient was seen for physical examination at the OPD. The mean time between e-TREPP and clinical follow-up for this study was 77 months (range: 25–130 months).

One patient had developed a late recurrence after 16 months, for which a re-TREPP was performed. 9 No re-recurrences were diagnosed. One patient ( *n* = 1) reported discomfort in the operated groin area described as not painful (VAS = 0). One patient ( *n* = 1) described a hypersensitivity in the operated area, but only during moments of increased abdominal pressure. Long-term follow-up after e-TREPP ( *n* = 10) revealed no presence of CPIP. The mean VAS-score at rest and VAS-score during activity were both zero, meaning no patient report pain in the inguinal area during rest or activities. There were no reports of discomfort during sexual activities. The Pain Disability Index (PDI 15 ) and the Short-Form 36 questionnaire (SF-36 16 ) showed no reported procedure-related disabilities.

It was considered to be inappropriate to perform a comparative or univariate regression analysis to identify risk factors for e-TREPP adverse events due to the small study population.

## Discussion

In this small case series, the safety and feasibility of e-TREPP for SIH repair were investigated. The e-TREPP appeared feasible in all cases and no conversion to other techniques was necessary. There was a low procedure-related complication rate and no patient complaints of CPIP at long-term follow-up. The outcomes on health-related quality of life, measured by the PDI and SF-36, showed encouraging results. The study results are limited due to the high number of patients lost to follow-up that had been deceased and the retrospective character.

In international guidelines, it is recommended to perform surgery immediately, in the case of suspected SIH. Neither type nor exact timing of the surgery is specified. Guidelines recommend to consider not to use a mesh in case of emergency repair in procedures with contaminated or dirty surgical area. 3 Only a limited number of studies report on this topic. In recent literature, however, mesh placement in emergency inguinal hernia repair seems to be a good option with acceptable wound infection rate and fewer recurrences than nonmesh repair (LoE 2A 6 ). 17 Another paper reported a prospective observational study with 234 patients where a prosthetic mesh was placed in SIH repair. During the follow-up of this study, 14 patients (6%) developed a wound infection, mesh infection occurred in one patient (0.5%), and the mesh was therefore removed. The authors concluded that the need for bowel resection is no contraindication for mesh placement (LoE 2B). 18 The same conclusion was drawn in a paper with results of a retrospective cross-sectional study that included 151 patients with incarcerated and/or strangulated (both are mentioned) inguinal hernia. In this paper, 112 patients underwent emergency surgical treatment with mesh-based repair, of which 15 needed bowel resections (LoE 3b). 19 In our study, from the two patients who developed an infection, one did undergo bowel surgery. Whether the preperitoneal approach and position of the mesh were safe for SIHs, such as the case in our series, was also evaluated in a randomized clinical trial focusing on the comparison between preperitoneal and Lichtenstein repair for strangulated groin hernias. 20 The results of this trial concluded that it was safe to use the preperitoneal approach, with the added benefit of a potential bowel resection without the need for an extra-midline incision (LoE 1D). 20 Another retrospective study gave the results of a different preperitoneal approach procedure in acute inguinal hernia repair (LoE 4). 21 In this retrospective study, 146 patients were included who underwent emergency surgery by open preperitoneal approach. A total of 15 patients (10.3%) of patients developed complications of which 6 patients (4.1%) developed a wound infection. None of the meshes had to be removed due to mesh infection. 21 Summarized, the limited literature in this field shows that both the preperitoneal approach and the use of synthetic mesh in SIH repair seem feasible with complication rates acceptable for emergency surgery.

Although these first e-TREPP results seem to be promising, we interpreted the results cautiously. We are aware of all methodological shortcomings of this first evaluation of e-TREPP (such as sample size, low long-term follow-up, and bias). Our research group surgeons are experts in the field of hernia repair. More research is needed to confirm or reject the first promising results of e-TREPP presented in this paper.

## Conclusion

e-TREPP in experienced hands seems feasible and safe (Level of Evidence 4) for the treatment of patients with strangulated inguinal hernia, with percentages of postoperative complications comparable to other techniques.
